# Case report: Usefulness of a picosecond Alexandrite laser therapy on atypical henna-induced Riehl's melanosis inferred from immunohistochemical analyses

**DOI:** 10.3389/fmed.2024.1401938

**Published:** 2024-06-11

**Authors:** Mami Kishimoto, Takanori Iwayama, Nobuyuki Horita, Takeshi Fukumoto

**Affiliations:** ^1^Division of Dermatology, Department of Internal Related, Kobe University Graduate School of Medicine, Kobe, Japan; ^2^Department of Dermatology, Kobe Ekisaikai Hospital, Kobe, Japan; ^3^Department of Plastic Surgery, Shinbian Total Skin Clinic, Osaka, Japan; ^4^Chemotherapy Center, Yokohama City University Hospital, Yokohama, Japan

**Keywords:** melanosis, henna, CD8 lymphocytes +, picosecond (ps) laser source, pathogenesis

## Abstract

Riehl's melanosis is a pigmented dermatitis that manifests as brown-gray facial pigmentation with pigment incontinence and infiltration of cells in the upper dermis. The associated inflammation is induced by a variety of products such as drugs and cosmetics. Henna, commonly referred to as a hypoallergenic cosmetic, has been reported to cause Riehl's melanosis in some cases. Although skin depigmenting agents have been occasionally used, satisfactory results have not been obtained and no established therapeutic strategies exist to treat Riehl's melanosis. Meanwhile, picosecond lasers effectively treat other hyperpigmentation disorders. In this study, we report safe and effective treatment of henna induced-atypical Riehl's melanosis using a 755-nm picosecond Alexandrite laser. Immunohistochemical analyses revealed a potential role of CD8-positive lymphocytes in henna-induced inflammation and hyperpigmentation of the basal layer, and a role of melanophages in the pigmented dermis of Riehl's melanosis.

Riehl's melanosis is a pigmented dermatitis that manifests as brown-gray facial pigmentation with pigment incontinence and infiltration of cells, namely melanophages and lymphocytes, in the upper dermis (1–3). The associated inflammation is triggered by a variety of products such as drugs and cosmetics (1). Henna is a known hypoallergenic cosmetic; however, some cases of henna-induced Riehl's melanosis have been reported (2).

Although skin depigmenting agents (such as hydroquinone, tretinoin, and vitamin C) have been occasionally used, satisfactory results have not been obtained and no established therapeutic strategies exist to treat Riehl's melanosis (1). Meanwhile, it is well-known that picosecond lasers effectively treat hyperpigmentation disorders (4, 5). In this case study, we report safe and effective treatment of henna-induced atypical Riehl's melanosis with a 755-nm picosecond Alexandrite laser and provide an insight into participating cells using immunohistochemical analyses.

The case study reports presentation of diffuse hyperpigmented patches all over face of a 78-yar-old female (Figures 1A–C). Prior to her first use of a henna-containing hair dye, skin patch tests for henna-containing hair dyes had been negative. No abnormalities were observed during the first 4 years of use; however, hyperpigmented patches gradually appeared on the face. The biopsy of her lower jaw revealed multiple necrotic keratinocytes and mild liquefaction degeneration in the basal layer, with lymphocytes as the main inflammatory cells (Figures 1D, E). Fontana–Masson staining confirmed the presence of melanin (Figures 1F, G). CD-68 positivity of melanin-containing dermal cells (Figures 1H, I) and CD-8 positivity of the inflammatory cells in the basal layer (Figures 1J, K) were found. Skin patch tests with henna-containing hair dyes and cosmetics showed positive reactions (Figures 1L–N).

**Figure 1:**
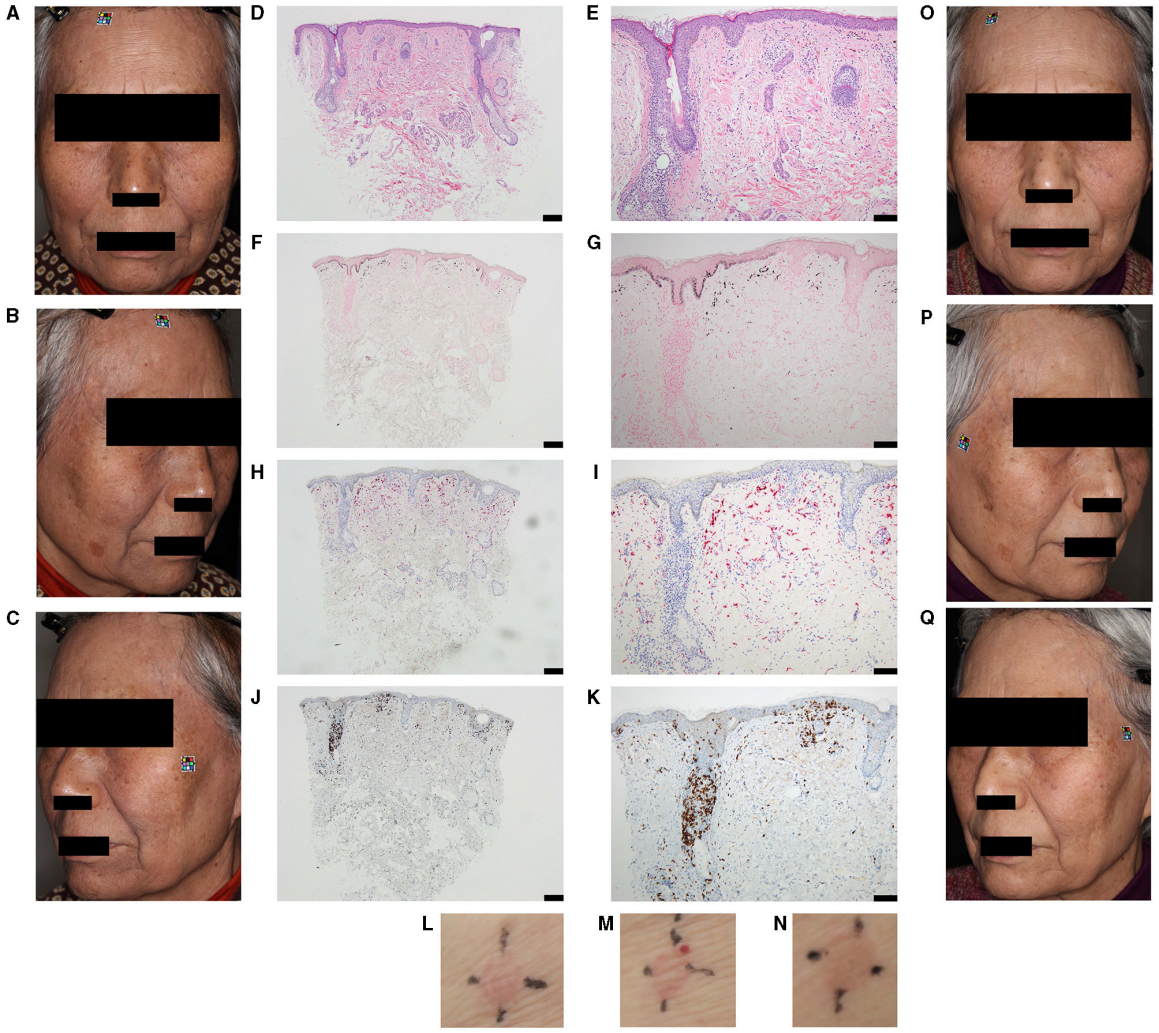
Clinical and histopathological features. **(A–C)** Diffuse brown-gray pigmentation over the entire face of the 78-year-old female patient is visible. Pigmentation on the most affected regions i.e., forehead, temples, and neck: **(A)** the front; **(B)** right side; **(C)** left side of the face. **(D–K)** Histopathological features of the skin of a hyperpigmented lesion on the lower jaw are shown. **(D, E)** Hematoxylin and eosin staining of the biopsy sample revealing pigmentary incontinence with infiltration of melanophages, lymphocytes, and a few eosinophils into the upper dermis; **(F, G)** Fontana–Masson staining confirms the presence of melanin; **(H, I)** CD-68 positive staining confirms the presence of macrophages in the upper dermis; **(J, K)** CD-8 positive staining confirms the presence of inflammatory cells in the basal layer. Magnification and scale bar of 100X and 200 μm **(D, F, H, J)** and 200X and 100 μm **(E, G, I, K)**, respectively. **(L–N)** Skin patch tests with henna-containing hair dyes and cosmetics confirm positive reactions. Positive reaction is seen for **(L)** liquid cleansing, **(M)** facial wash, and **(N)** hair dye at 72 h. **(O–Q)** Ameliorated pigmentation after thirteen sessions of irradiation of the patient's entire face, **(O)** the front; **(P)** right side; **(Q)** left side, with a 755-nm picosecond Alexandrite laser is shown.

Next, we discussed the possibility that this case was caused by other drugs. The only drugs used before the onset of Riehl's melanosis were inhalation of vilanterol trifenatate and fluticasone furoate. To the best of our knowledge, there were no reports of inhalation of vilanterol trifenatate and fluticasone furoate causing hyperpigmentation only on the face. Moreover, no topical products were used externally other than the cosmetics tested in the patch test.

Finally, henna-induced atypical Riehl's melanosis was diagnosed. As initial treatments with skin depigmenting agents were unsuccessful, a picosecond Alexandrite laser therapy (PicoSure PSAL; Cynosure, Westfood, MA, USA) was performed every 4 weeks for a total of 13 sessions, till the pigmentation visibly improved (Figures 1O–Q). The irradiation parameters were as follows: Fluence was gradually increased by 0.25 (1st−4th) to 0.40 (5th−7th), and 0.71 (8th−13th) J/cm^2^ with a pulse width of 750 ps. Immunohistochemical analyses revealed a potential role of CD8-positive lymphocytes in henna-induced inflammation and hyperpigmentation of the basal layer, and role of melanophages in the pigmented dermis in Riehl's melanosis pathology.

Typical hair dye-induced Riehl's melanosis mainly occurs in the lateral and upper surfaces of the face (1, 2), but the current patient had atypical hyperpigmentation of the entire face. It is suspected that the lesions spread to the entire face due to the usage of henna-containing facial cosmetic products as well as hair dye. Henna is known as an ingredient in hair dyes, but it is also found in cosmetics.

Although this case study demonstrated that CD8-positive T cells may be involved in the pathogenesis of Riehl's melanosis, the detailed role of CD8-positive T cells in Riehl's melanosis has not been evaluated. However, based on previous reports in other diseases (6), a similar mechanism may occur, in which CD8-positive T cells attack the epidermal basal layer, resulting in liquefaction degeneration in the basal layer and pigment incontinence.

We think that picosecond alexandrite lasers are suitable for the treatment of Riehl's melanosis because they selectively and efficiently destroy melanosomes in the epidermis with minimal damage to the surrounding tissue (3). To the best of our knowledge, there are no reports on whether picosecond alexandrite lasers can also have any effect on CD8-positive T cells in Riehl's melanosis. On the other hand, it has been reported that NB-UVB treatment affects T cells in the epidermis, reducing the proportion of pathogenic T cells via inducing apoptosis (7–9). The effect of picosecond alexandrite laser on T cells needs to be further investigated.

## Data availability statement

The original contributions presented in the study are included in the article/supplementary material, further inquiries can be directed to the corresponding author.

## Ethics statement

The studies involving humans were approved by the Ethics Committee of Kobe University. The studies were conducted in accordance with the local legislation and institutional requirements. The participants provided their written informed consent to participate in this study. Written informed consent was obtained from the individual(s) for the publication of any potentially identifiable images or data included in this article.

## Author contributions

MK: Data curation, Formal analysis, Investigation, Methodology, Project administration, Software, Validation, Writing – original draft, Writing – review & editing. TI: Data curation, Formal analysis, Investigation, Methodology, Software, Conceptualization, Project administration, Validation, Writing – review & editing, Supervision. NH: Conceptualization, Data curation, Formal analysis, Investigation, Methodology, Project administration, Validation, Writing – review & editing, Software, Supervision. TF: Conceptualization, Data curation, Formal analysis, Investigation, Methodology, Software, Supervision, Writing – review & editing, Funding acquisition, Project administration, Resources, Validation, Visualization, Writing – original draft.
